# Metastasis of Thyroid Cancer to the Sternum after Total Thyroidectomy and Laryngectomy

**DOI:** 10.1155/2013/346246

**Published:** 2013-09-12

**Authors:** Hajime Ishinaga, Tomotaka Miyamura, Hironori Tenpaku, Kazuhiko Takeuchi

**Affiliations:** ^1^Department of Otorhinolaryngology-Head and Neck Surgery, Mie University Graduate School of Medicine, 2-174 Edobashi, Tsu, Mie 514-8507, Japan; ^2^Department of Thoracic and Cardiovascular Surgery, Mie University Graduate School of Medicine, 2-174 Edobashi, Tsu, Mie 514-8507, Japan

## Abstract

Metastasis of thyroid cancer to the sternum is rare. Ablation is the therapy of choice for patients with metastasizing differentiated thyroid cancer, while surgical resection is an option for those with resectable bony metastasis. This report describes a case of a 65-year-old woman with a sternal tumor. The patient was treated by partial sternal resection and sternal reconstruction with new material polypropylene/expanded polytetrafluoroethylene (ePTFE) composite. The postoperative course was uneventful, and she was free of recurrence after 1 year of follow-up. We conclude that surgery should be used to manage solid bony metastasis from thyroid papillary carcinoma. Further more, a polypropylene/ePTFE composite may be useful for sternal reconstruction after thoracotomy.

## 1. Introduction

Sternal metastasis from intrathoracic or extrathoracic malignancy is rare. Most sternal metastases arise from breast cancer, and only a few cases of sternal metastasis arising from papillary carcinoma have been reported [1]. Though ablation is the therapy of choice for patients with metastasizing differentiated thyroid cancer, patients with metastases to the bones have poor rates of survival when compared with those metastases to the cervical lymph nodes and lung [2]. Therefore, surgical resection is recommended for patients with resectable bony metastasis. The present report describes a case of sternal metastasis from papillary adenocarcinoma of the thyroid gland that was treated by partial sternal resection and sternal reconstruction with a polypropylene/ePTFE composite.

## 2. Case Report

A 65-year-old woman was admitted to Mie University Hospital for management of a sternal tumor. She had undergone left-hemithyroidectomy with a left-modified neck dissection for papillary adenocarcinoma (T3N1bM0) in our department 9 years earlier. Four years after the first operation, she had undergone right-hemithyroidectomy with right-modified neck dissection at another hospital because of locoregional recurrence. She returned to our hospital 2 years ago because computed tomography (CT) demonstrated recurrence with invasion into the thyroid and cricoid cartilage. Therefore, total laryngectomy was performed.

**Figure 1:**
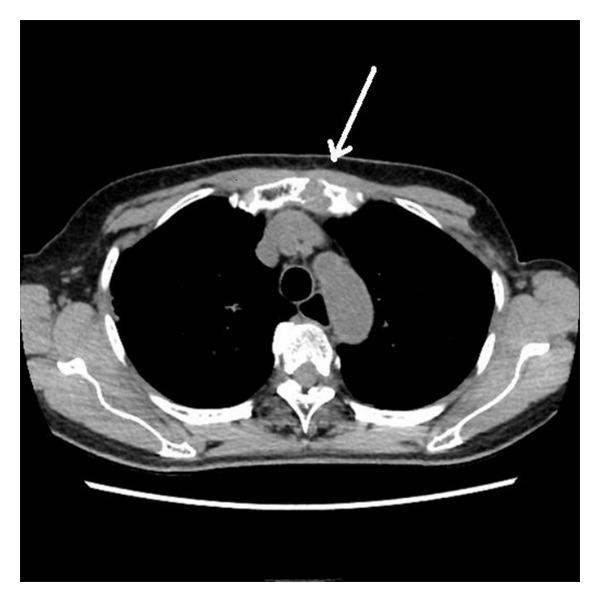
Computed tomography scans of the mass. The arrow indicates the sternal metastasis.

On examination, the patient was euthyroid in the context of exogenous thyroid hormone administration. A permanent tracheostomy was present and there was no mass palpable on the anterior chest wall. No lymph node enlargement was noted either in the neck or the mediastinum, and no pulmonary metastases were found. CT demonstrated a soft-tissue mass originating from the upper sternum (Figure 1) measuring 20 × 20 mm. No invasion of the tumor into the thoracic cavity was found. FDG-PET/CT scan demonstrated intense FDG uptake only in the sternum. Although radioiodine scan showed no apparent uptake in the sternum, a CT scan-guided tru-cut biopsy revealed tissue with features that were typical of metastatic papillary adenocarcinoma of the thyroid.

**Figure 2:**
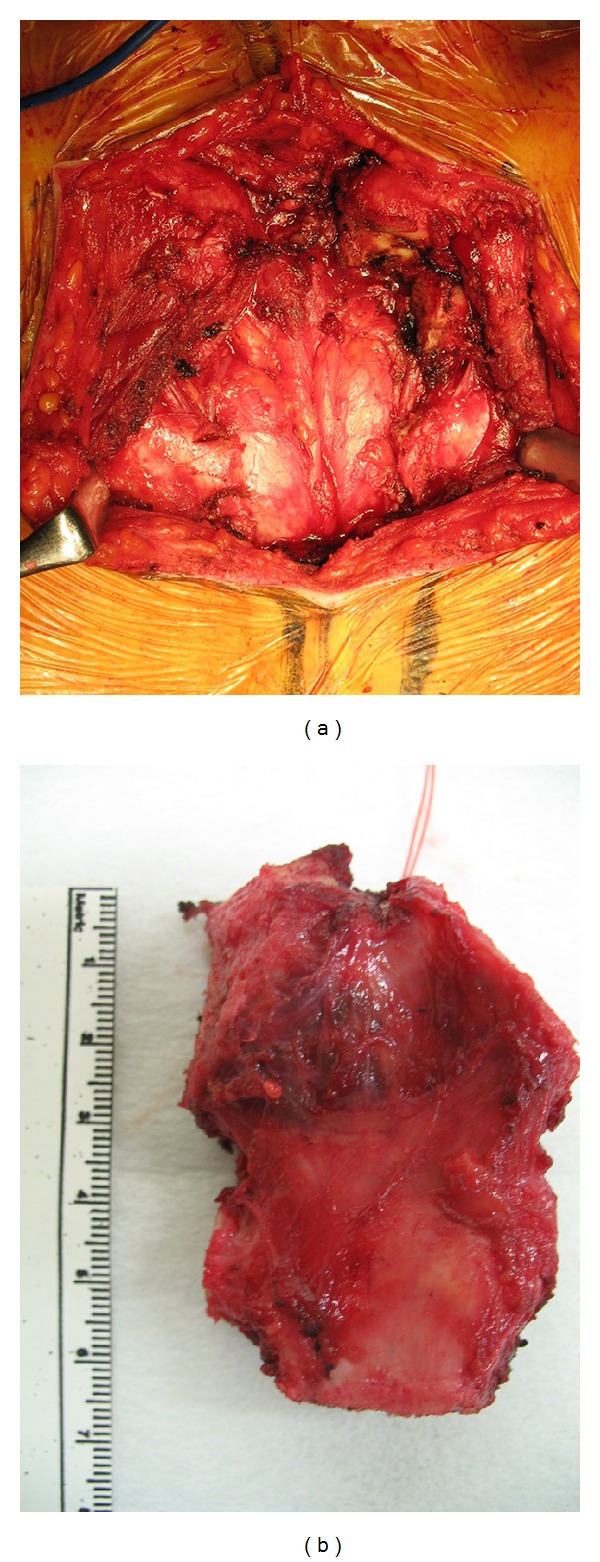
Intra-operative photography after resection of the sternal metastasis (a) and surgical specimen (b).

**Figure 3:**
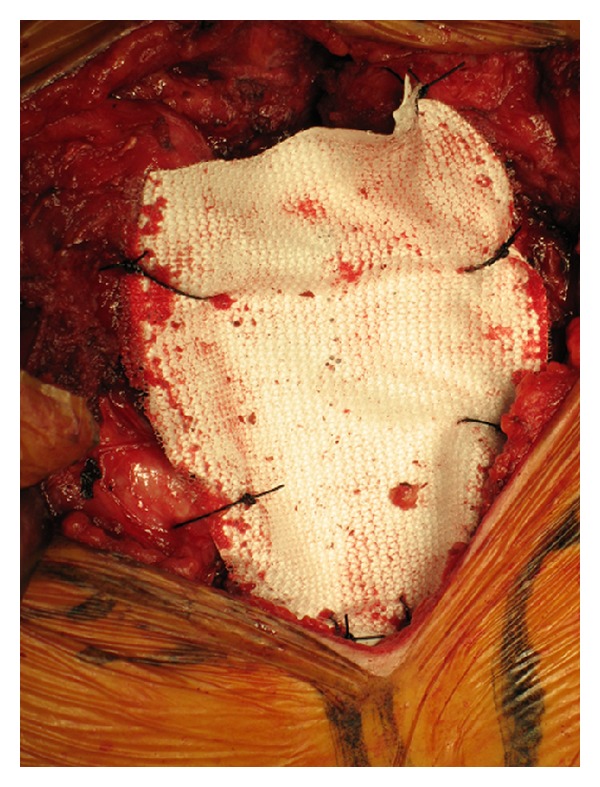
Repair of the sternal defect using a polypropylene/ePTFE composite.

At operation, a T-shaped skin incision was made over the tumor, preserving the permanent tracheostomy. The sternum was cut horizontally at the level of the third costochondral junction and the first and second ribs (Figure 2). Negative margins were observed on frozen section analysis. Sternal reconstruction was performed with a polypropylene/ePTFE composite, which had been sutured with 0-Prolene to the edges of the resected chest wall (Figure 3). Then, pectoralis major muscle was sutured to cover the mesh and to reinforce the anterior chest. Pathologic analysis of the surgical specimens revealed papillary adenocarcinoma. The patient was discharged on the tenth postoperative day on levothyroxine-suppression therapy. She had no physical or pulmonary impairment and no evidence of tumor recurrence on CT or by serum thyroglobulin level at follow-up 14 months after surgery. And this reconstruction showed a stabilized chest wall.

## 3. Discussion

Distant metastasis occurs in 10–20% of cases of well-differentiated thyroid carcinoma, mostly in the lung and bone [3]. Bone metastasis (BM) is diagnosed clinically in 4–23% of patients with differentiated thyroid carcinoma and is associated with poor prognosis [4]. In the current patient of well-differentiated thyroid carcinoma population, the disease-specific survival rate from diagnosis of initial BM was 36% at 5 years and was 10% at 10 years [5].

In general, radioactive iodine (RAI) therapy is the gold standard for the treatment of distant metastasis from thyroid cancer. Good candidates for RAI therapy include younger patients and those with well-differentiated tumor, high-RAI uptake, small metastases, and stable or slow disease. However, bone metastase are associated with worse prognoses and they do not respond well to radioiodine ablation. Therefore, surgery should be performed in the case of solitary bone metastasis. Zettinig et al. reported that surgical extirpation of bone metastases was associated with improved survival in a subgroup of patients with distant metastases limited to the bones [4]. This approach may provide better outcomes with an improved quality of life in the case of isolated metastases that are amenable to resection [4]. Yanagawa et al. reported that 10 cases of sternal thyroid cancer metastasis had relatively good outcomes following treatment by partial sternectomy and RAI in 5 patients [1]. In our case, the patient had single bone metastasis within the sternum, and this patient was expected to have good outcomes in response to complete surgical resection. Therefore, partial sternectomy was performed in our department. 

Many reports have described closure and coverage after resection of cranial sternum. The defect should be covered either by autogenous or artificial substitutes. Autoplastic reconstruction is indicated for smaller defects, while alloplastic materials are indicated for larger defects. Several studies have reported good results in response to reconstruction with skin flap, myocutaneous flaps, Marlex mesh, Gore-Tex dual mesh, methyl methacrylate sandwiched between Marlex mesh, steel mesh to titanium plate, and so on [1, 6, 7]. In the present case, reconstruction was performed with a polypropylene/ePTFE composite, which is originally used for the reconstruction of the abdominal wall [8]. Polypropylene mesh was used on one side to promote tissue ingrowth, while submicronic ePTFE was used on the other side to minimize adhesions to the prosthesis [8]. This approach achieved good aesthetic and functional results in this case.

In conclusion, sternal metastasis from thyroid carcinoma is not common. Complete resection may provide disease-free survival with good quality of life. A polypropylene/ePTFE composite may be advantageous for reconstruction of the sternum.

## References

[1] Yanagawa J, Abtin F, Lai CK (2009). Resection of thyroid cancer metastases to the sternum. *Journal of Thoracic Oncology*.

[2] Meyer A, Behrend M (2005). Partial resection of the sternum for osseous metastasis of differentiated thyroid cancer: case report. *Anticancer Research*.

[3] Leger AF (1995). Distant metastases of differentiated thyroid cancer. Diagnosis by 131 iodine (I 131) and treatment. *Annales d’Endocrinologie*.

[4] Zettinig G, Fueger BJ, Passler C (2002). Long-term follow-up of patients with bone metastases from differentiated thyroid carcinoma—surgery or conventional therapy?. *Clinical Endocrinology*.

[5] Orita Y, Sugitani I, Matsuura M (2010). Prognostic factors and the therapeutic strategy for patients with bone metastasis from differentiated thyroid carcinoma. *Surgery*.

[6] Haraguchi S, Yamashita Y, Yamashita K, Hioki M, Matsumoto K, Shimizu K (2004). Sternal resection for metastasis from thyroid carcinoma and reconstruction with the sandwiched marlex and stainless steel mesh. *Japanese Journal of Thoracic and Cardiovascular Surgery*.

[7] Moraitis S, Perelas A, Toufektzian L, Mazarakis N, Pechlivanides G (2012). Giant sternal metastasis secondary to follicular carcinoma of the thyroid gland: report of a case. *Surgery Today*.

[8] Cobb WS, Harris JB, Lokey JS, Mcgill ES, Klove KL (2003). Incisional herniorrhaphy with intraperitoneal composite mesh: a report of 95 cases. *The American Surgeon*.

